# Optical Diffraction in Close Proximity to Plane Apertures. IV. Test of a Pseudo-Vectorial Theory

**DOI:** 10.6028/jres.111.001

**Published:** 2006-02-01

**Authors:** Klaus D. Mielenz

**Affiliations:** National Institute of Standards and Technology, Gaithersburg, MD 20899-8440

**Keywords:** diffraction, narrow slits, optics, polarization, pseudo-vectorial theory, small circular apertures, scalar theory, transmission coefficients

## Abstract

Rayleigh’s pseudo-vectorial theory of the diffraction of polarized light by apertures which are small compared to the wavelength of light is analyzed with respect to its mathematical rigor and physical significance. It is found that the results published by Rayleigh and Bouwkamp for *s*-polarized incident do not obey the conditions assumed in their derivation and must therefore be dismissed. It is also found that the theory leads to paradoxical predictions concerning the polarization of the diffracted field, so that the pseudo-vectorial approach is intrinsically incapable of describing polarization effects.

## 1. Introduction

**Fig. 1:**
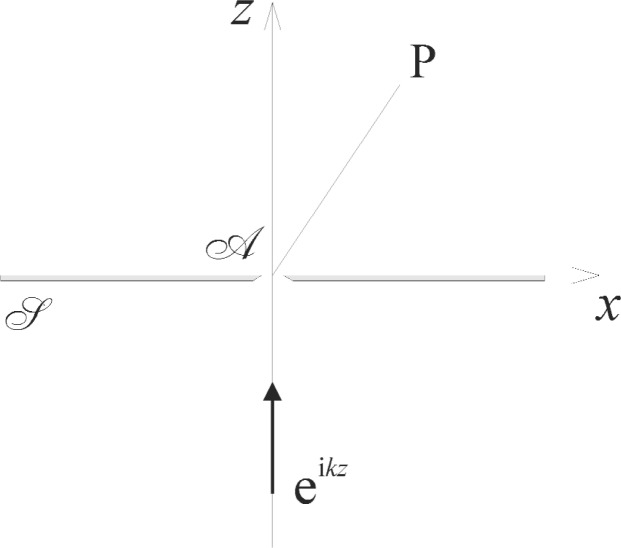
The Rayleigh-Bouwkamp diffraction problem. A very narrow aperture A in a metallic screen S which is located in the *xy*-plane is illuminated by a monochromatic, *p*- or *s*-polarized plane wave e^i^*^kz^*, and solutions are constructed so that the optical field is everywhere continuous with continuous first derivatives.

**Fig. 2:**
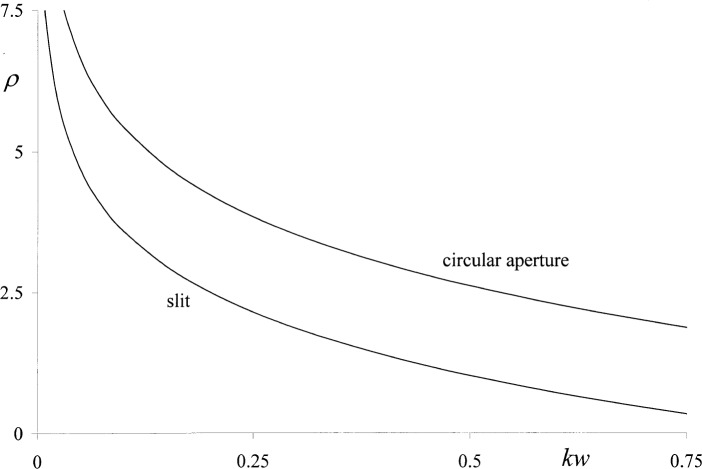
Polarization ratios, $\rho =\tau_{\text{RB}}^{\left(p\right)}/\tau_{\text{RB}}^{\left(s\right)}$, of circular apertures and slits vs. aperture size *kw* according to Bouwkamp [3, p. 71ff].

Most applications of diffraction theory in optics are based on the scalar approximation of Huygens’ principle, and it is usually understood that polarization is ignored. A notable exception to this practice is the classical pseudo-vectorial theory formulated by Rayleigh [1,2] and Bouwkamp [3] for the diffraction of plane-polarized light by small metallic apertures as illustrated in Fig. 1. The results obtained by these authors implied large polarization effects in the case of aperture sizes smaller than the wavelength of light, *λ*. Specifically, Rayleigh and Bouwkamp predicted that unpolarized light diffracted by very narrow aperture will become strongly *p*-polarized, as indicated by the ratio $\tau_{\text{RB}}^{\left(p\right)}/\tau_{\text{RB}}^{\left(s\right)}$ of the transmission coefficients of circular apertures and slits illuminated by normally incident *p*- or s-polarized light plotted in Fig. 2 for the range 0 < *kw* ≤ 0.75, where 2*w* is the aperture width and *k* = 2π/*λ* is the circular wave number. Regarding the case of slits, Rayleigh commented: “It appeared that if the width of the slit is very small in comparison with the wavelength, there is a much more free passage when the electric vector is perpendicular to the slit than when it is parallel to the slit, so that unpolarized light incident upon the screen will, after passage, appear polarized in the former manner.” While this may be plausible as far as slits are concerned, it seems impossible that a circular aperture can behave in the same manner because normal incidence is assumed so that symmetry would dictate a complete absence of polarization. Apparently Rayleigh had similar doubts. He remarked that the *p*-solution has “no simple application” in the case of circular apertures, but nonetheless the discrepancy is disturbing as the Rayleigh-Bouwkamp theory is commonly referred to as a “rigorous” theory, and in this connection it should also be noted that later authors, such as Levine and Schwinger [4,5], considered only the *s*-solution and, in doing so, made no reference to polarization. In the present paper the Rayleigh-Bouwkamp theory will be reexamined with respect to its physical significance, mathematical rigor, and accuracy.

Rayleigh considered a monochromatic plane wave of unit irradiance, *u*_0_ = e^i^*^kz^*, which is incident from the half space *z* < 0 on a circular aperture or slit A contained in an infinite, infinitesimally thin, opaque screen S that lies in the *xy*-plane of a cartesian coordinate system (Fig. 1). He also assumed that the diffracted field in the half space *z* ≥ 0 obeys the Rayleigh-Sommerfeld integral equations,

$$u^{\left(p\right)}\left(P\right)=-\frac{1}{2\pi }\int_{A}\text{dQ}\frac{\partial u^{\left(p\right)}\left(Q\right)}{\partial z}\frac{e^{ikQP}}{QP},\;z\geq 0,$$ (1a)

$$u^{\left(s\right)}\left(P\right)=-\frac{1}{2\pi }\int_{A}\text{dQ}u^{\left(s\right)}\left(Q\right)\frac{\partial }{\partial z}\left(\frac{e^{ikQP}}{QP}\right),\;z\geq 0,$$ (1b)

where Q = (*ξ*, *η*, 0) is a point in A, P = (*x*, *y*, *z*) is an arbitrary point in space, *QP* is the distance between them, *u*^(^*^p,s^*^)^ are scalar wave functions, and *∂ u*^(^*^p^*^)^(*Q*)/*∂ z* and *u*^(^*^s^*^)^(*Q*) are zero on the opaque portion of S as if the screen is a perfect metallic reflector and the incident light is *p*- or *s*-polarized. Furthermore, Rayleigh assumed that field on the source side of S is a modified geometrical field of the form

$$u^{\left(p,s\right)}\left(P\right)=e^{ikz}\pm e^{-ikz}+u_{-}^{\left(p,s\right)}\left(P\right),\;z\leq 0,$$ (1c)

and hence the objective of the theory is to find the unknown quantities *u*^(^*^p,s^*^)^(*P*) and $u_{-}^{\left(p,s\right)}\left(P\right)$ so that the overall field is continuously differentiable in the aperture plane.

From Eq. (1c) it follows that *∂u*^(^*^p^*^)^/*∂z* and *u*^(^*^s^*^)^ will be continuous for *z* = 0 if

$$\frac{\partial u^{\left(p\right)}\left(x,y,0\right)}{\partial z}=\frac{\partial u_{-}^{\left(p\right)}\left(x,y,0\right)}{\partial z},u^{\left(s\right)}\left(x,y,0\right)=u_{-}^{\left(s\right)}\left(x,y,0\right),$$ (2a)

so that

$$u_{-}^{\left(p,s\right)}\left(x,y,z\right)=\mp u^{\left(p,s\right)}\left(x,y,-z\right),\;z\geq 0,$$ (2b)

$$u^{\left(p,s\right)}\left(x,y,z\right)=e^{ikz}\pm e^{-ikz}\mp u^{\left(p,s\right)}\left(x,y,-z\right),\;z\geq 0.$$ (2c)

Thus, *u*^(^*^p^*^)^ = 2 − *u*^(^*^p^*^)^, ∂*u*^(^*^s^*^)^/∂*z* = 2i*k* − ∂*u*^(^*^s^*^)^/∂*z* when *z* = 0, so that *u*^(^*^p^*^)^ and ∂*u*^(^*^s^*^)^/∂*z* will also be continuous if

$$u^{\left(p\right)}\left(x,y,0\right)=1,\frac{1}{ik}\frac{\partial u^{\left(s\right)}\left(x,y,0\right)}{\partial z}=1$$ (2d)

The conditions [Eq. (2d)] represent the underlying integral equations of the Rayleigh-Bouwkamp and other so-called “rigorous” diffraction theories which are intended to solve the wave equation in the presence of prescribed boundary conditions. To this author’s knowledge, all existing solutions with the sole exception of Sommerfeld’s half-plane theory are approximations of some sort and in the cases considered here these approximations take the form of ascending power series in *kw* which were derived on the assumption that the unknown aperture distributions in Eqs. (1a,b) can be expressed in the form

$$\begin{array}{l} \frac{1}{ik}\frac{\partial u^{\left(p\right)}\left(Q\right)}{\partial z}=-\frac{i}{kw}\left[\frac{p_{0}}{W}+{\left(kw\right)}^{2}p_{1}W+\ldots \right],\\ u^{\left(s\right)}\left(Q\right)=-ikw\left[s_{1}W+{\left(kw\right)}^{2}s_{2}W^{3}+\ldots \right], \end{array}$$ (3)

where $W=\sqrt{1-{\left(q/w\right)}^{2}}$, *q* is the distance of *Q* from the coordinate origin, and the coefficients *p_n_* and *s_n_* are to be chosen so that Eqs. (2d) are satisfied. The determination of these coefficients is tedious and will not be repeated here as the results have been detailed in Ref. [3]. In the following the corresponding solutions will be denoted by the subscript ‘RB’ and it will be sufficient to consider only the initial terms of the expansions [Eq. (3)] by assuming, as Rayleigh did originally, that *kw* → 0.

## 2. Circular Apertures

For circular apertures it is appropriate to use polar coordinates inside the aperture and in the plane of observation, so that

$$P=\left(\rho \;\cos\varphi ,\rho \;\sin\varphi ,\;z\right),\;\rho^{2}=x^{2}+y^{2},$$ (4a)

$$Q=\left(q\;\cos\chi ,q\;\sin\chi ,\;0\right),\;q^{2}=\xi^{2}+\eta^{2},$$ (4b)

$$\text{dQ}=qdqd\chi ,\;QP^{2}=z^{2}+\rho^{2}+q^{2}-2\rho q\cos\left(\varphi -\chi \right).$$ (4c)

The initial terms of the trial solutions of Eq. (3) were determined by Rayleigh and Bouwkamp as *p*_0_ = *s*_1_ = −2/π and thus the first-order solutions for circular apertures are

$$u_{\text{RB}}^{\left(p\right)}\left(P\right)=\frac{1}{\pi^{2}}\int_{0}^{w}dq\int_{0}^{2ð}d\chi \frac{qe^{ikQP}}{\sqrt{w^{2}-q^{2}}QP},\;kw\to 0,\;z\geq 0,$$ (4d)

$$\begin{array}{l} u_{\text{RB}}^{\left(s\right)}\left(P\right)=-\frac{ik}{\pi^{2}}\int_{0}^{w}dq\int_{0}^{2ð}d\chi q\sqrt{w^{2}-q^{2}}\frac{\partial }{\partial z}\left(\frac{e^{ikQP}}{QP}\right)=\frac{k^{2}z}{\pi^{2}}\\ \int_{0}^{w}dq\int_{0}^{2ð}d\chi q\sqrt{w^{2}-q^{2}}e^{ikQP}\left(\frac{1}{QP^{2}}+\frac{i}{k\;QP^{3}}\right),kw\to 0,\;z\geq 0. \end{array}$$ (4e)

### 2.1 Fresnel Approximation

The evaluation of Eqs. (4d,e) in the Fresnel limit is simple. When *z* ≫ *λ*, and thus *z* >> *q*, the dependence of *QP* on the aperture coordinates can be ignored, so that

$$QP∼\sqrt{z^{2}+\rho^{2}}=r,\;\frac{z}{r}=\cos\theta$$ (5a)

and

$$u_{\text{RB}}^{\left(p\right)}\left(P\right)=-\frac{w}{\pi^{2}}\frac{e^{ikr}}{r}\int_{0}^{1}\frac{d\left(q/w\right)\left(q/w\right)}{\sqrt{1-{\left(q/w\right)}^{2}}}\int_{0}^{2\pi }d\chi =\frac{2we^{ikr}}{\pi r},\;z\gg \lambda ,$$ (5b)

$$\begin{array}{l} u_{\text{RB}}^{\left(s\right)}\left(P\right)=\frac{2k^{2}w^{3}\cos\theta e^{ikr}}{\pi r}\int_{0}^{1}d\left(q/w\right)\left(q/w\right)\sqrt{1-{\left(q/w\right)}^{2}},\\ \;=\frac{2k^{2}w^{3}\cos\theta e^{ikr}}{3\pi r},\;z\gg \lambda \end{array}$$ (5c)

where *q*/*w* = sin*α* was substituted in both integrals. Hence, the total radiant fluxes transmitted by the aperture and incident on the inside of a large hemisphere with radius *r* are given by

$$\Phi^{\left(p\right)}=2\pi r^{2}\int_{0}^{\pi /2}d\theta \sin\theta {\left|u_{\text{RB}}^{\left(p\right)}\right|}^{2}=\frac{8w^{2}}{\pi },$$ (5d)

$$\begin{array}{l} \Phi^{\left(s\right)}=2\pi r^{2}\int_{0}^{\pi /2}d\theta \sin\theta {\left|u_{\text{RB}}^{\left(s\right)}\right|}^{2}=\frac{8k^{4}w^{6}}{9\pi }\int_{0}^{\pi /2}d\theta \sin\theta \cos^{2}\theta \\ \;=\frac{8k^{4}w^{6}}{27\pi }, \end{array}$$ (5e)

and the corresponding transmission coefficients of the aperture are

$$\tau_{\text{RB}}^{\left(p\right)}=\frac{\Phi^{\left(s\right)}}{\Phi_{0}}=\frac{8}{\pi^{2}},\;\tau_{\text{RB}}^{\left(s\right)}=\frac{\Phi^{\left(s\right)}}{\Phi_{0}}=\frac{8{\left(kw\right)}^{4}}{27\pi^{2}},\;kw\to 0,$$ (5f)

where Φ_0_= π*w*^2^ is the geometrical flux incident on the aperture.

These results can now be checked for consistency with Eqs. (2d) and (3) by using an alternative expression [3,6] for transmission coefficients,

$$\tau^{\prime }=\frac{1}{A}\int_{A}dQ{\left|u\left(Q\right)\right|}^{2},$$ (6a)

where *A* is the area of the aperture and *u*(Q) is the field inside it. According to Eq. (2d) we expect $u_{\text{RB}}^{\left(p\right)}\left(Q\right)=1$ and therefore

$${\left(\tau_{\text{RB}}^{\left(p\right)}\right)}^{\prime }=1,$$ (6b)

whereas from Eq. (3) and *s*_1_ = −2/π it follows that

$${\left(\tau_{\text{RB}}^{\left(s\right)}\right)}^{\prime }=\frac{{\left(s_{1}kw\right)}^{2}}{\pi w^{2}}\int_{0}^{2\pi }d\chi \int_{0}^{1}dqq\left[1-{\left(q/w\right)}^{2}\right]=\frac{2{\left(kw\right)}^{2}}{\pi },\;kw\to 0.$$ (6c)

The *p*-coefficients of Eqs. (5f) and (6b) agree reasonably well. On the other hand, there is a considerable difference between the corresponding *s*-coefficients.

### 2.2 Near-Field Solution

In the immediate proximity of the aperture plane the exponential e^i^*^kQP^* is effectively constant and equal to 1 inside the aperture, and Eqs. (4d,e) can be simplified as follows,

$$u_{\text{RB}}^{\left(p\right)}\left(P\right)=\frac{1}{\pi^{2}}\int_{0}^{w}\frac{qdq}{\sqrt{w^{2}-q^{2}}}\int_{0}^{2\pi }\frac{d\chi }{QP},\;z\ll \lambda$$ (7a)

$$u_{\text{RB}}^{\left(s\right)}\left(P\right)=-\frac{k^{2}z}{\pi^{2}}\int_{0}^{w}dqq\sqrt{w^{2}-q^{2}}\int_{0}^{2\pi }d\chi \left(\frac{1}{QP^{2}}+\frac{i}{kQP^{3}}\right),\;z\ll \lambda .$$ (7b)

As these wave functions must be rotationally symmetrical about the *z*-axis it suffices to evaluate the *χ*-integrals appearing in these expressions for *φ* = 0. Substituting *α* = (*χ* − π)/2, we find

$$QP^{2}=z^{2}+\rho^{2}+q^{2}-2\rho q\cos\chi =\frac{4\rho q}{m}\left(1-m\sin^{2}\alpha \right),$$ (8a)

$$m=\frac{4\rho q}{z^{2}+{\left(\rho +q\right)}^{2}}$$ (8b)

and hence

$$\int_{0}^{2\pi }\frac{d\chi }{QP}=2\sqrt{\frac{m}{\rho q}}\int_{0}^{\pi /2}\frac{d\alpha }{{\left(1-m\sin^{2}\alpha \right)}^{1/2}}=2\sqrt{\frac{m}{\rho q}}K\left(m\right),$$ (8c)

$$\int_{0}^{2\pi }\frac{d\chi }{QP^{2}}=\frac{m}{\rho q}\int_{0}^{\pi /2}\frac{d\alpha }{1-m\sin^{2}\alpha }=\frac{\pi m}{2\rho q\sqrt{1-m}},$$ (8d)

$$\int_{0}^{2\pi }\frac{d\chi }{QP^{3}}=\frac{1}{2}{\left(\frac{m}{\rho q}\right)}^{3/2}\int_{0}^{\pi /2}\frac{d\alpha }{{\left(1-m\sin^{2}\alpha \right)}^{3/2}}=\frac{1}{2}{\left(\frac{m}{\rho q}\right)}^{3/2}\frac{E\left(m\right)}{1-m},$$ (8e)

where K(*m*) and E(*m*) are the complete elliptical integrals of the first and second kinds [7,8].

Upon substitution of Eq. (8c), Eq. (7a) is reduced to the single integral

$$u_{\text{RB}}^{\left(p\right)}\left(P\right)=\frac{2}{\pi^{2}\sqrt{\rho /w}}\int_{0}^{1}\frac{d\left(q/w\right)\sqrt{mq/w}K\left(m\right)}{\sqrt{1-{\left(q/w\right)}^{2}}},\;z\ll \lambda ,$$ (9a)

where relative coordinates are used. This expression was evaluated by numerical integration to analyze its behavior in the aperture plane, using a polynomial approximation for K(*m*) given in Ref. [7] and assuming *z*/*w* = 0 as well as *z*/*w* = 0.01 in order to find $\partial u_{\text{RB}}^{\left(p\right)}/\partial z$ by numerical differentiation. The results of these computations are listed in the left-hand portion of Table 1, which shows that they are in satisfactory agreement with the values expected from Eqs. (2d) and (4d).

The corresponding expressions for $u_{\text{RB}}^{\left(s\right)}$ are obtained from Eqs. (7b) and (8d) and can be expressed as follows,

$$\begin{array}{l} u_{\text{RB}}^{\left(s\right)}\left(P\right)=kz\left[\frac{kwA}{\rho /w}+\frac{iB}{{\left(\rho /w\right)}^{3/2}}\right],\frac{1}{ik}\frac{\partial u_{\text{RB}}^{\left(s\right)}\left(P\right)}{\partial z}\\ \;=\frac{B}{{\left(\rho /w\right)}^{3/2}}-\frac{iA}{\rho /w}. \end{array}$$ (10a)

where

$$A=-\frac{1}{2\pi }\int_{0}^{1}\frac{d\left(q/w\right)m\sqrt{1-{\left(q/w\right)}^{2}}}{\sqrt{1-m}},\;z\ll \lambda ,$$ (10b)

$$B=-\frac{1}{2\pi^{2}}\int_{0}^{1}\frac{d\left(q/w\right)m^{3/2}\sqrt{1-{\left(q/w\right)}^{2}}E\left(m\right)}{\left(1-m\right)\sqrt{q/w}},\;z\ll \lambda ,$$ (10c)

and *E*(*m*) can also be expressed by a polynomial approximation [7]. It was ascertained that the integrals *A* and *B* are everywhere finite, and hence it follows from the first Eq. (10a) that the aperture values of $u_{\text{RB}}^{\left(s\right)}$ are identically equal to zero. This is in disagreement with Eq. (4e), and it was also found that the computed aperture values of $\left(1/ik\right)\partial u_{\text{RB}}^{\left(s\right)}/\partial z$ do not satisfy the second integral equation, [Eq. (2d)]. These discrepancies are documented in the right-hand portion of Table 1.

## 3. Slits

The second case considered by Rayleigh and Bouwkamp is that of an infinitely long, narrow slit of width 2*w* which is centered on the *y*-axis of a rectangular coordinate system. As the resulting diffraction pattern will be independent of *y*, it is sufficient to compute its variation in the *xz*-plane by assuming

P=(x,0,z),Q=(ξ,η,0), (11a)

and then Eqs. (1a,b) are reduced to

$$\begin{array}{l} u_{\text{RB}}^{\left(p\right)}\left(P\right)=-\frac{1}{2\pi }\int_{-w}^{w}d\xi \frac{\partial u_{\text{RB}}^{\left(p\right)}\left(\xi ,0\right)}{\partial z}\int_{-\infty }^{\infty }d\eta \frac{e^{ik\sqrt{{\left(\xi -x\right)}^{2}+\eta^{2}+z^{2}}}}{\sqrt{{\left(\xi -x\right)}^{2}+\eta^{2}+z^{2}}}\\ =-\frac{i}{2}\int_{-w}^{w}d\xi \frac{\partial u_{\text{RB}}^{\left(p\right)}\left(\xi ,0\right)}{\partial z}H_{0}^{\left(1\right)}\left(\beta \right),\beta =k\sqrt{{\left(\xi -x\right)}^{2}+z^{2}}, \end{array}$$ (11b)

$$\begin{array}{l} u_{\text{RB}}^{\left(p\right)}\left(P\right)=-\frac{i}{2}\int_{-w}^{w}d\xi u_{\text{RB}}^{\left(s\right)}\left(\xi ,0\right)\frac{\partial H_{0}^{\left(1\right)}\left(\beta \right)}{\partial z}\\ =-\frac{ik^{2}z}{2}\int_{-w}^{w}d\xi u_{\text{RB}}^{\left(s\right)}\left(\xi ,0\right)\frac{H_{1}^{\left(1\right)}\left(\beta \right)}{\beta }, \end{array}$$ (11c)

where the infinite *η*-integral in Eq. (10b) was evaluated in terms of the Hankel function $H_{0}^{\left(1\right)}\left(\beta \right)$ [8] and the relationship ${\text{dH}}_{0}^{\left(1\right)}\left(\beta \right)/d\beta =-H_{1}^{\left(1\right)}\beta$ was used in Eq. (10c). According to Rayleigh and Bouwkamp the initial terms of the series expansions [Eq. (3)] are *p*_0_ = 1/(*o* − iπ/2) and *s*_1_ = 1 where and *o* = ln(*γkw*/4) and *γ* = 0.577.. is Euler’s constant, and hence the first-order solutions for slits are

$$u_{\text{RB}}^{\left(p\right)}\left(P\right)=-\frac{ip_{0}}{2w}\int_{-w}^{w}\frac{d\xi H_{0}^{\left(1\right)}\left(\beta \right)}{\sqrt{1-{\left(\xi /w\right)}^{2}}},\;kw\to 0,\;z\geq 0,$$ (11d)

$$u_{\text{RB}}^{\left(p\right)}\left(P\right)=-\frac{k^{3}wz}{2}\int_{-w}^{w}\frac{d\xi \sqrt{1-{\left(\xi /w\right)}^{2}}H_{1}^{\left(1\right)}\left(\beta \right)}{\beta },\;kw\to 0,\;z\geq 0,$$ (11e)

### 3.1 Fresnel Approximation

At large distances from the slit (*z* ≫ *w*), the phase difference *β* defined in Eq. (11b) is effectively a large constant and equal to $kr=k\sqrt{x^{2}+z^{2}}$, and the Hankel functions in Eqs. (11d,e) can be replaced by their asymptotic expansions

$$H_{0}^{\left(1\right)}\left(\beta \right)∼\sqrt{\frac{2}{\pi kr}}e^{i\left(kr-\pi /4\right)},\frac{H_{0}^{\left(1\right)}\beta }{\beta }∼\sqrt{\frac{2}{\pi {\left(kr\right)}^{3}}}e^{i\left(kr-3\pi /4\right)},$$ (12a)

so that

$$\begin{array}{l} u_{\text{RB}}^{\left(p\right)}\left(P\right)=-\frac{i\sqrt{2}p_{0}e^{i\left(kr-\pi /4\right)}}{\sqrt{\pi kr}}\int_{0}^{1}\frac{d\left(\xi /w\right)}{\sqrt{1-{\left(\xi /w\right)}^{2}}}\\ \;=-\frac{i\sqrt{\pi }e^{i\left(kr-\pi /4\right)}}{\left(o-i\pi /2\right)\sqrt{2kr}},\;z\gg \lambda , \end{array}$$ (12b)

$$\begin{array}{l} u_{\text{RB}}^{\left(s\right)}\left(P\right)=-\frac{\sqrt{2}{\left(kw\right)}^{2}\cos\theta e^{i\left(kr-3\pi /4\right)}}{\sqrt{\pi kr}}\int_{0}^{1}d\left(\xi /w\right)\sqrt{1-{\left(\xi /w\right)}^{2}}\\ \;=-\frac{\sqrt{\pi }{\left(kw\right)}^{2}\cos\theta e^{i\left(kr-3\pi /4\right)}}{\sqrt{8kr}},\;z\gg \lambda , \end{array}$$ (12c)

where cos*θ* = *z*/*r* and the two integrals were again evaluated by substituting *ξ*/*w* = sin*α*. These expressions can now be used to compute the quantities

$$\Delta \Phi^{\left(p,s\right)}=r\Delta y\int_{-\pi /2}^{\pi /2}d\theta {\left|u_{RB}^{\left(p,s\right)}\right|}^{2},$$ (12d)

which represent the radiant flux emerging from a slit segment of length Δ*y* and falling on the corresponding annular segment of a semicylinder of radius *r* which is centered on the *y*-axis. As the geometrical flux incident on the slit segment is ΔΦ_0_ = 2*w* Δ*y*, this leads at once to the following expressions for the transmission coefficients of the slit,

$$\begin{array}{l} \tau_{\text{RB}}^{\left(p\right)}=\frac{\Delta \Phi^{\left(p\right)}}{\Delta \Phi_{0}}=\frac{\pi^{2}{\left|p_{0}\right|}^{2}}{4kw}=\frac{\pi^{2}/4kw}{o^{2}+\pi^{2}/4},\;\tau_{\text{RB}}^{\left(s\right)}=\frac{\Delta \Phi^{\left(s\right)}}{\Delta \Phi_{0}}\\ \;=\frac{\pi^{2}{\left(kw\right)}^{3}}{32},\;kw\to 0. \end{array}$$ (12e)

As in Sec. 2.1 alternative expressions for these transmission coefficients can be obtained from Eq. (6a), which in this case is equivalent to

$$\tau^{\prime }=\frac{1}{w}\int_{0}^{w}d\xi {\left|u\left(\xi \right)\right|}^{2}.$$ (13a)

Hence we find, using Eqs. (2d) and (3) with *s*_1_ = 1,

$${\left(\tau_{\text{RB}}^{\left(p\right)}\right)}^{\prime }=1,$$ (13b)

$${\left(\tau_{\text{RB}}^{\left(s\right)}\right)}^{\prime }=kw\int_{0}^{w}d\xi \left[1-{\left(\xi /w\right)}^{2}\right]=\frac{2{\left(kw\right)}^{2}}{3},\;kw\to 0.$$ (13c)

Both of these results disagree with the corresponding values in Eq. (12e).

### 3.2 Near-Field Solution

The integrals [Eq. (11d,e)] can be evaluated without simplifying assumptions for arbitrary values of *z*, and it was found that they are finite in the aperture plane in spite of the singularities of $H_{0}^{\left(1\right)}\left(\beta \right)$ and $H_{1}^{\left(1\right)}\left(\beta \right)/\beta$ for *β* = 0.

For *p*-polarized light we find

$$\begin{array}{l} u_{\text{RB}}^{\left(p\right)}\left(P\right)\\ =\frac{1}{2M}\int_{-1}^{1}\frac{d\left(\xi /w\right)\left\{\left[\pi J_{0}\left(\beta \right)/2+oY_{0}\left(\beta \right)\right]-i\left[oJ_{0}\left(\beta \right)-\pi Y_{0}\left(\beta \right)/2\right]\right\}}{\sqrt{1-{\left(\xi /w\right)}^{2}}}, \end{array}$$ (14)

where J_n_ and Y_n_ are Bessel functions of the first and second kinds and *M* = *o*^2^ + π^2^/4. The integral [Eq. (14)] was evaluated numerically for *kw* = 0.1 and *z* = 0 as well as *z* = 0.01*w*, yielding the results for $u_{\text{RB}}^{\left(p\right)}$ and $\partial u_{\text{RB}}^{\left(p\right)}/\partial z$ shown in the left-hand portion of Table 2. The table also shows the corresponding values expected from Eqs. (2d) and (3), and the agreement was judged satisfactory in view of the approximations made in Rayleigh’s derivations.

The corresponding expression obtained from Eq. (11e) is

$$u_{\text{RB}}^{\left(s\right)}\left(P\right)=-\frac{k^{2}wz}{2}\left(C+iD\right),$$ (15a)

where

$$\begin{array}{l} C=\int_{-1}^{1}\frac{d\left(\xi /w\right)\sqrt{1-{\left(\xi /w\right)}^{2}J_{1}\left(\beta \right)}}{\beta },\\ \;D=\int_{-1}^{1}\frac{d\left(\xi /w\right)\sqrt{1-{\left(\xi /w\right)}^{2}Y_{1}\left(\beta \right)}}{\beta }. \end{array}$$ (15b)

It was found that the integrals [Eq. (15b)] are finite in the aperture plane, so that according to Eq. (15a) the aperture values of $u_{\text{RB}}^{\left(s\right)}$ RB are identically zero and thus do not agree with the assumed values of Eq. (3). Equation (15a) also shows that

$$\frac{1}{ik}\frac{\partial u_{\text{RB}}^{\left(s\right)}\left(P\right)}{\partial z}=\frac{kw}{2}\left(D-iC\right),\;\text{if}\;z=0,$$ (15c)

which obviously disagrees with Eq. (2d). The various numerical results obtained for $u_{\text{RB}}^{\left(s\right)}$ and $\partial u_{\text{RB}}^{\left(s\right)}/\partial z$ are listed on the right-hand side of Table 2.

## 4. Conclusion

Based on the above-mentioned results and illustrated by the numerical examples shown in Tables 1 and 2 there can be no doubt that the Rayleigh-Bouwkamp theory is mathematically flawed and fails to give a credible account of the diffraction of polarized light. The reasons for this conclusion are twofold.

The near-field solutions derived in Secs 2.2 and 3.2 for *p*-polarized incident light $\left(\partial u_{0}/\partial z=0\text{on}S\right)$ are in approximate agreement with the conditions in Eqs. (2d) and (3) that were assumed in their derivation, and thus appear to be free of contradictions. On the other hand, the corresponding solutions for *s*-polarization $\left(u_{0}=0\;\text{on}\;S\right)$ do not obey these conditions at all. This discrepancy was found for circular apertures and slits alike, and thus points to a general problem associated with the second integral equation [Eq. (2d)] as such. Bouwkamp [3] mentioned that this integral equation [Eq. (2d)] is notoriously difficult as it requires differentiation with respect to *z* under the integral sign and can lead to divergent solutions. He pointed out that these divergencies can be eliminated, but this was not attempted in this work. In any case, the polarization effects shown in Fig. 1 must be dismissed as wrong.

A second, and most likely uncorrectable, problem arises from the fact that the ‘polarization effects’ predicted by the Rayleigh-Bouwkamp theory run counter to common experience. As mentioned in the Introduction, a polarized field behind a circular aperture illuminated by normally incident, unpolarized light is impossible for reasons of symmetry. Yet the existence of such a field is implied by the upper curve in Fig. 1, and the discrepancy persists even when the suspect transmission coefficient $\tau_{\text{RB}}^{\left(s\right)}$ in Eq. (5f) is replaced by the improved coefficient ${\left(\tau_{\text{RB}}^{\left(s\right)}\right)}^{\prime }$ in Eq. (6c). Additionally, the different values obtained in Secs. 2.1 and 3.1 for the far- and near-field transmission coefficients seemed paradoxical because the polarization of light cannot change during free-space propagation. As this discrepancy was encountered for both states of polarization, it also cannot be blamed on the mathematical problem mentioned in the previous paragraph.

Hence it is concluded that the Rayleigh-Bouwkamp theory cannot be relied upon for describing the diffraction of polarized light by small apertures. Virtually the same determination was made in an earlier paper [6] on the feasibility of using the Rayleigh-Sommerfeld boundary-value integrals for analyzing diffraction by apertures of arbitrary size, and thus the overall conclusion is that the inability to account for polarization effects is an inherent property of scalar diffraction theories, no matter what boundary conditions are assumed. The fact of the matter appears to be that, in scalar diffraction theory, the boundary conditions ∂*u*_0_/∂*z* = 0 or *u*_0_ = 0 are necessary and sufficient to satisfy Helmholtz’ theorem. As it happens, the same conditions also pertain to the metallic reflection of *p*- or *s*-polarized light but it does not follow that they are sufficient to ensure conformance with electromagnetic theory.

In Ref. [6] it was also shown that a modified theory of unpolarized diffraction can be formulated in which the Rayleigh-Sommerfeld integrals are used to describe a continuous, bidirectional flow of energy in the near zones on either side of an aperture. The transmission coefficients computed in this manner are similar to those published by Levine and Schwinger [4,5] for subwavelength aperture sizes, so that a special theory for this region may not be needed.

## Figures and Tables

**Table 1:** Aperture values of wave functions and normal derivatives for circular apertures of width *kw* = 0.1, as computed from Eqs. (9a) and (10a,b,c) and expected from Eqs. (2d) and (3)

*ρ*/*w*	$u_{\text{RB}}^{\left(p\right)}\left(Q\right)$		$\left(1/ik\right)\partial u_{\text{RB}}^{\left(p\right)}\left(Q\right)/\partial z$		$u_{\text{RB}}^{\left(s\right)}\left(Q\right)$		$\left(1/ik\right)\partial u_{\text{RB}}^{\left(s\right)}\left(Q\right)/\partial z$	
	computed	expected	computed	expected	computed	expected	computed	expected
0.01	0.99	1	−0.62	−0.66	0	0.064 i	0.5 i	1
0.25	0.99	1	−0.64	−0.66	0	0.062 i	−30 + 0.5 i	1
0.50	0.99	1	−0.72	−0.74	0	0.055 i	−216 + 0.4 i	1
0.75	0.99	1	−0.94	−0.96	0	0.042 i	−558 + 0.3 i	1
0.99	0.98	1	−3.91	−4.51	0	0.009 i	−276	1

**Table 2:** Aperture values of wave functions and normal derivatives for slits of width *kw* = 0.1, as computed from Eqs. (14) and (15a,b,c) and expected from Eqs. (2d) and (3)

*ρ*/*w*	$u_{\text{RB}}^{\left(p\right)}\left(Q\right)$		$\left(1/ik\right)\partial u_{\text{RB}}^{\left(p\right)}\left(Q\right)/\partial z$		$u_{\text{RB}}^{\left(s\right)}\left(Q\right)$		$\left(1/ik\right)\partial u_{\text{RB}}^{\left(s\right)}\left(Q\right)/\partial z$	
	computed	expected	computed	expected	computed	expected	computed	expected
0.01	0.76	1	0.75 + 2.0 i	0.76 + 2.1 i	0	−0.10 i	8.6 – 38 i	1
0.25	0.76	1	0.78 + 2.1 i	0.79 + 2.1 i	0	−0.10 i	8.3 – 37 i	1
0.50	0.76	1	0.87 + 2.3 i	0.89 + 2.4 i	0	−0.09 i	7.4 – 33 i	1
0.75	0.76	1	1.1 + 3.1 i	1.2 + 3.1 i	0	−0.05 i	5.8 – 25 i	1
0.99	0.75	1	4.7 + 13 i	5.5 + 15 i	0	−0.01 i	1.6 – 4 i	1
